# Fatality of Small Pox at Different Ages

**Published:** 1871-04

**Authors:** 


Fatality of Small-pox at Different Ages.—The Registrar-General states that of 755 deaths registered in London from small-pox during the last five weeks, 348 occurred to children under five years of age, 196 to young people aged between five and twenty years, 168 to persons between the ages of twenty and forty, and 43 to persons over forty years of age. Taking the ages of the living into account, the mortality in the five weeks from small-pox was at the annual rate of 10·6 per 1,000 among young children, of 2·6 per 1,000 between the ages of five and twenty, of 2·1 per 1,000 between twenty and forty, and of less than 1 per 1000 above forty years of age. It is thus shown that the fatality of the disease is four times as great in the first five years of life as it is at any subsequent period, and we hope that the knowledge of this fact will be spread abroad as affording indisputable proof of the importance of early vaccination.

